# VANGUARD®crLyme: A next generation Lyme disease vaccine that prevents *B. burgdorferi* infection in dogs

**DOI:** 10.1016/j.jvacx.2020.100079

**Published:** 2020-10-09

**Authors:** Richard T. Marconi, David Garcia-Tapia, Jacquelien Hoevers, Nicole Honsberger, Vickie L. King, Dianne Ritter, Denise J. Schwahn, Leroy Swearingin, Angela Weber, M. Teresa C. Winkler, Jason Millership

**Affiliations:** a Department of Microbiology and Immunology, Virginia Commonwealth University Medical Center, Richmond, VA 23298-0678, United States; b Zoetis Inc., 333 Portage Road, Kalamazoo, MI 49007-4931, United States

**Keywords:** Borrelia, Lyme disease, vaccine, crLyme, OspC, OspA, Borreliella, chimeritope

## Abstract

Lyme disease, a public health threat of significance to both veterinary and human medicine, is caused by the tick (*Ixodes*) transmitted spirochete, *Borreliella burgdorferi*. Here we report on the immunogenicity and efficacy of VANGUARD®crLyme (Zoetis), the most recent canine Lyme disease vaccine to be approved by the United States Department of Agriculture. VANGUARD®crLyme is a subunit vaccine consisting of outer surface protein A (OspA) and a recombinant outer surface protein C (OspC) based-chimeric epitope protein (chimeritope) that consists of at least 14 different linear epitopes derived from diverse OspC proteins. The combination of OspA and the OspC chimeritope (Ch14) in the vaccine formulation allows for the development of humoral immune responses that work synergistically to target spirochetes in both ticks and in mammals. Immunogenicity was assessed in purpose-bred dogs. A two-dose vaccination protocol resulted in high antibody titers to OspA and Ch14 and vaccinal antibody reacted with 25 different recombinant OspC variants. Efficacy was demonstrated using an *Ixodes scapularis* -purpose bred dog challenge model. Vaccination with VANGUARD®crLyme provided protection against infection and prevented the development of clinical manifestations and histopathological changes associated with Lyme disease.

## 1 Introduction

Lyme disease (LD) is the most common vector-borne disease in the northern hemisphere [1], [2]. The etiological agent, a spirochete, was identified in humans in 1983 [3], assigned to the genus *Borrelia* and named *Borrelia burgdorferi* [4]. Shortly thereafter, Lyme arthritis of spirochetal etiology was reported in canines [5]. Based on several independent genetic analyses, *B. burgdorferi* was divided into multiple species [6], [7], [8], [9]. *B. burgdorferi* is the primary causative agent of LD in North America. *B. burgdorferi, B. afzelli, B. garinii,* and *B. bavariensis* are causative agents in Europe and Asia. Additional species have been identified-but have not been clearly associated with overt clinical disease. In 2014, the LD spirochetes were assigned separate genus status and classified as *Borreliella* [10].

The incidence of LD in humans and companion animals in N. America [11] and Europe [2] has been steadily rising. The most robust seroprevalence dataset for LD has come from routine serological screening of dogs. The Companion Animal Parasite Council (CAPC) reported 359,461 LD Ab (+) tests in 2019 in dogs in the US alone^1^. However, since results are reported for<30% of the total number of tests that are run each year, the actual number of Ab (+) tests may be closer to 1,000,000 per year. CAPC prevalence maps for LD correlate well with geographic regions with established or emerging *Ixodes* tick populations [12]. The LD spirochetes are maintained in nature in an enzootic cycle that involves *Ixodes* ticks and a diverse array of mammalian reservoirs [13]. In the eastern and western regions of North America, *Ixodes scapularis* and *I. pacificus* are the dominant tick vectors while *I. ricinus* and *I. persulcatus* dominate in Europe^2^.

Clinical manifestations of canine LD typically develop slowly and are initially non-descript [14], [15]. As LD progresses, intermittent lameness and polyarthritis become common [16]. Chronic infections can trigger protein-losing glomerulopathy leading to acute progressive renal failure [17], heart block [18], and neurological complications [19]. Sub-clinical LD has been demonstrated in laboratory infected canines by histopathology. Microlesions and inflammation of the tissues, synovial membranes, joint capsules, and associated tendon sheaths are common [20]. Hyperkeratosis, lymphoplasmacytic vasculitis, arteritis, perineuritis, meningitis, joint pannus, chronic suppurative arthritis, and glomerulitis may also develop. While infected dogs develop a robust Ab response to *B. burgdorferi* [16], [21], the humoral immune response is in most cases insufficient to clear an existing infection [22]. Protection that may develop from natural infection is typically short-lived and strain specific [23], [24].

Subunit and bacterin vaccines are available for the prevention of LD in dogs [25], [26]. Subunit vaccines are of defined composition consisting of one or two purified recombinant proteins while bacterins, which are whole cell lysates of two or more cultured LD spirochete strains, contain well in excess of 1,000 different proteins and protein variants. The overwhelming majority of proteins in bacterins are extraneous ingredients that have not been demonstrated to contribute to the development of protective Ab responses [27], [28]. In addition, bacterins lack numerous immunodominant antigens that are produced during infection in mammals or ticks but are not produced during cultivation [28]. As a case in point, the transcriptional expression rank of OspC is among the highest of all *B. burgdorferi* proteins in *I. scapularis* nymphs (8th) and mammals (1st) [28]. However, in cultured spirochetes its rank drops to 816 [28]. Conversely, the transcriptional expression rank of OspA is 8th during cultivation and 18th in larval ticks, respectively, but transcript is undetectable in LD spirochetes in mammals [28], [29]. Consistent with their enzootic cycle-stage specific expression patterns, OspC is essential for infection of mammals [30], [31] and OspA is required for survival in ticks [32]. OspA and OspC have been the primary focus of LD subunit vaccine development efforts (reviewed in [26]).

Distinct variants of OspC, referred to as OspC types (differentiated by letter or other designations), have been identified [33], [34], [35], [36]. The variable domains of OspC harbor the well-characterized L5 and H5 immunodominant epitopes [36]. Ab responses to OspC are largely ‘type’ specific [37], [38], [39] and are directed at L5 and H5. Conserved or ‘universal’ domains of OspC do not appear to contribute to protective Ab responses [38]. The conserved C-terminal residues of OspC (referred to as the C7 or C10 domain) have been suggested to elicit bactericidal Ab responses [40]. However, it has been demonstrated that recombinant OspC proteins lacking the C7/C10 domain are as effective as full length OspC in eliciting bactericidal antibody [38], [4]. The maintenance of antigenically distinct types of OspC in nature has been postulated to result in a balanced polymorphism of OspC types such that the LD spirochetes can infect reservoir populations that are immunologically primed from previous or ongoing infection with strains producing heterologous OspC types [41], [42]. The inherent diversity of OspC impeded early efforts to develop OspC as a vaccinogen [23], [37]. To overcome OspC diversity, chimeric epitope-based recombinant proteins referred to as chimeritopes were developed that consist of L5 and H5 epitopes derived from different OspC types. The immunogenicity of these unique proteins was initially assessed in mice and rats [43], [44], [45], [46]. OspC chimeritopes have been demonstrated to elicit Abs that recognize diverse OspC types [46]. VANGUARD®crLyme (Zoetis) canine LD vaccine, which is the subject of this report, consists of an OspC chimeritope (designated as Ch14) and OspA. Since the launch of VANGUARD®crLyme (Zoetis) in 2016, over 10.5 million doses of the vaccine have been distributed making it the most widely used LD vaccine in North America. Here we demonstrate that vaccination of dogs with VANGUARD®crLyme (Zoetis) provided protection against *B. burgdorferi* infection and prevented the development of clinical manifestations associated with LD.

## 2 Materials and methods

### 2.1 Animal procedures and vaccination protocols

**Fig. 1:**
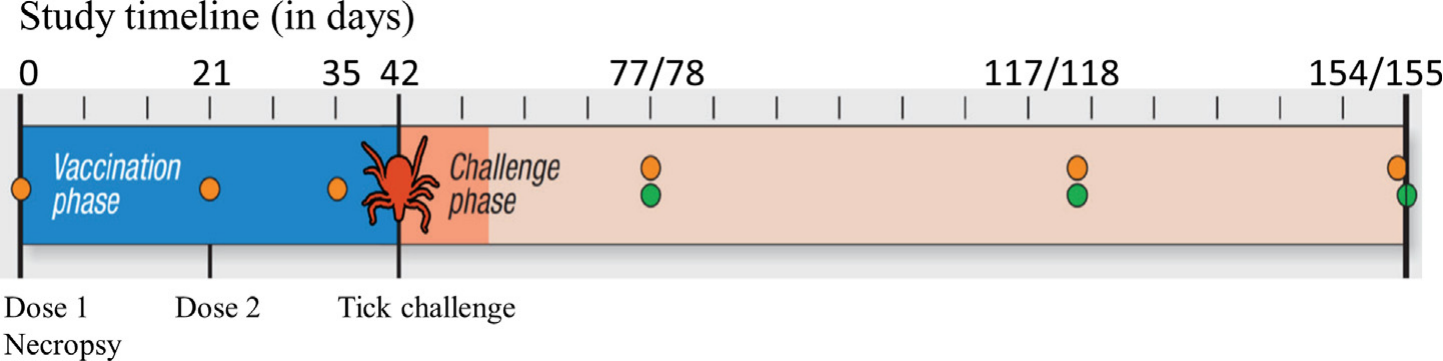
Schematic representation of study procedures and timeline. The schematic presented above indicates the timeline for significant procedures conducted in this study. The labels “dose 1” and “dose 2” indicate the days on which placebo or VANGUARD®crLyme vaccine were delivered. Blood draws (Days 0, 21, 35, 77, 117 and 153) and tissue biopsy (Days 78, 118 and 154/155) events are indicated by orange and green circles, respectively. Necropsies were performed over two days (154/155) to obtain samples for histopathology. The study also included four sentinel dogs that were not exposed to placebo, vaccine, or infected ticks but were otherwise assessed as all other study dogs. (For interpretation of the references to colour in this figure legend, the reader is referred to the web version of this article.)

Antibody profile defined laboratory-purposed beagles (7.1 to 7.7 weeks of age; 18 male/18 female; Ridglan Farms) were obtained and randomly allocated to treatment groups, rooms, and pens at the study site using a statistical software suite (SAS Institute). Dogs were maintained at Zoetis research sites in accordance with USDA Animal Welfare Regulations (9 Code of Federal Regulations, Chapter 1, Subchapter A – Animal Welfare) and approved Institutional Animal Care and Use Committee (IACUC) protocols (Zoetis). Prophylactic vaccines for parvovirus and *Bordetella bronchiseptica* were administered to all study dogs. Four dogs were randomly selected to be sentinels (no additional vaccination or exposure to ticks), 16 dogs were assigned to the T01 placebo group (PBS with adjuvant; henceforth referred to as ‘placebo dogs’) and 16 were assigned to the T02 vaccine group (henceforth referred to as ‘vaccinate dogs’). VANGUARD®crLyme (Zoetis), henceforth referred to as ‘vaccine’, was manufactured using practices in accordance with USDA-approved Outline of Production (proprietary information, data not shown) at the minimum immunizing dose. Placebo and vaccine were administered on Day 0 (approximately 8-week-old dogs; subcutaneous injection; right dorsal scapular area) and Day 21 (subcutaneous injection; left dorsal scapular area). On Day 0, dogs were tested for Ab to the *B. burgdorferi* C6 peptide using the SNAP® 4Dx Test (IDEXX). Note that subsequent C6 Ab analyses conducted in this study employed the SNAP4Dx® Plus Test (IDEXX). After each treatment, dogs were closely monitored for adverse events as detailed below. A study timeline is presented in Fig. 1.

### 2.2 Tick infestation and challenge analyses

Tick challenge was performed using adult *I. scapularis* ticks collected from a collection site in Wakefield, Rhode Island (latitude and longitude: 41.438171 and −71.501556) (fee for service; Dr. Thomas Mather). Prior to infestation, ticks were stored in vented vials (12–15 °C; 90–95% humidity) and the percentage of *B. burgdorferi* infected ticks (56.7%) determined by PCR using *B. burgdorferi flaB* specific primers. On Day 42, 30 ticks per dog (15 male/15 female) were placed in infestation chambers, placed on placebo and vaccinate dogs, and the ticks were fed to repletion. The ticks were collected on study Day 51. For the remaining course of the study, the dogs were monitored for clinical signs of LD including ataxia, lameness, depression, lethargy, head pressing, myalgia, and other manifestations.

### 2.3 Immunological analyses

Blood was collected from each dog on Days 0, 21, 35, 77, 117, and 153, and sera harvested using standard methods. The SNAP® 4Dx and SNAP4®Dx Plus tests were performed as instructed by the manufacturer (IDEXX). ELISA assays for OspA and Ch14 were performed using standard methods. In brief, detection antigens (250 ng; OspA and Ch14) were immobilized onto 96-well microtiter plates (overnight; 4 °C; 0.01 M Borate buffer), the plates contents were removed and the wells were washed with blocking buffer (PBS, 1% Casein, 0.05% Tween 20®; 37 °C; 60 min) followed by a final wash with PBST (PBS, 0.05% Tween 20). Serially diluted sera were added and the plates were incubated at 37 °C for 1 hr. After washing, horseradish peroxidase conjugated goat anti-dog IgG(H + L) was added (1:1000; 37 °C; 1 hr), the plates were washed, ABTS substrate was added (≤15 min; RT) and absorbance read at A_405-490_. ELISAs were run in duplicate and antigen specific titers calculated from the average plus three standard deviations of the negative control A_405-490_ value. The reciprocal test sample dilution above the average negative control OD represented the endpoint titer. Vaccination induced antibody responses to diverse OspC type proteins were assessed by immunoblot using recombinant OspC proteins exactly as decribed in an earlier study [46].

### 2.4 Histopathology

Tissue from the area of the tick feeding site, left and right (L/R) shoulder synovial joint tissue, L/R elbow synovial joint tissue, L/R carpus synovial joint tissue, L/R stifle synovial joint tissue, L/R tarsus synovial joint tissue, kidneys, femur, and humerus were collected. Samples were blinded and then formalin fixed, processed, embedded, sectioned, mounted, and stained with hematoxylin and eosin on a fee for service basis by an independent party (Charles River). Sections were examined by a blinded board-certified Veterinary Pathologist (Zoetis Inc.) using light microscopy. A severity grade assessing the type, distribution, and severity of inflammation was assigned based on a scoring system where 0 = absent, 1 = minimal, 2 = mild, 3 = moderate, 4 = marked, and 5 = severe; scores >= 2 were considered abnormal.

### 2.5 Statistical analysis

All hypothesis tests were carried out at the 0.05 level of significance (two sided; *P* ≤ 0.05). Data were analyzed using SAS® software (SAS Inc, Cary, NC). A Cochran-Armitage test adjusting for post-infestation room was used to analyze C6 Ab ever being positive post-tick challenge, having at least one joint graded 2 or higher, and the presentation of disease.

## 3 Results

### 3.1 Vaccination, tick infestation and clinical assessment

Dogs were administered placebo or vaccine without complication. Ticks successfully fed on all placebos and vaccinates. Neither injection site swelling nor pain were observed in any of the placebos or vaccinates. Lameness was observed in 3/16 (18.75%) placebo dogs at one or more timepoints post-tick feeding (Table 1). No clinical indicators of distress or LD infection were observed in the sentinel or vaccinates. Placebo dogs that developed lameness were treated with Metacam® (Meloxicam; Boehringer Ingelheim VetMedica Inc.), a non-steroidal anti-inflammatory medication.

**Table 1:** Data summary.

Group	Episodic Lameness	*B. burgdorferi*C6 Ab + SNAP4Dx PLUS	Abnormal Histology^a^	Disease Positive
**Placebo**	3/16 (18.75%)	16/16 (100%)	13/16 (81.3%)	16/16 (100%)
**Vaccinates**	0/16 (0%)	1/16 (6.25%) *P ≤* 0.0001	0/16 (0%) *P ≤* 0.0001	0/16 (0%) *P ≤* 0.0001
**Sentinel**	0/4 (0%)	0/4 (0%)	0/4 (0%)	0/4 (0%)

a Inflammation graded 2 or higher in at least one joint.

### 3.2 Analysis of Ab responses pre- and post-vaccination

All sentinel dogs were C6 Ab (-) at all timepoints. All placebo dogs (16/16) were C6 Ab (+) on two or more occasions post-tick feeding. Seven of sixteen placebo dogs were C6 Ab (+) on Day 77 and all were (+) on Days 117 and 153. Fifteen of sixteen vaccinates were C6 Ab (-) at all timepoints. A single vaccinate (dog WAR2) was C6 Ab (+) on Day 77, but (-) on Days 117 and 153. In total, of the 48 C6 Ab tests conducted per study group after tick infestation, 97.9% and 18.8% of the tests done on vaccinate and placebo dogs were (-), respectively. Results are summarized in Table 1. It can be concluded the placebo dogs were successfully infected with *B. burgdorferi* and that vaccination with VANGUARD®crLyme resulted in a significant difference in the prevention of *B. burgdorferi* infection compared to placebo (*P* < 0.0001).

**Fig. 2:**
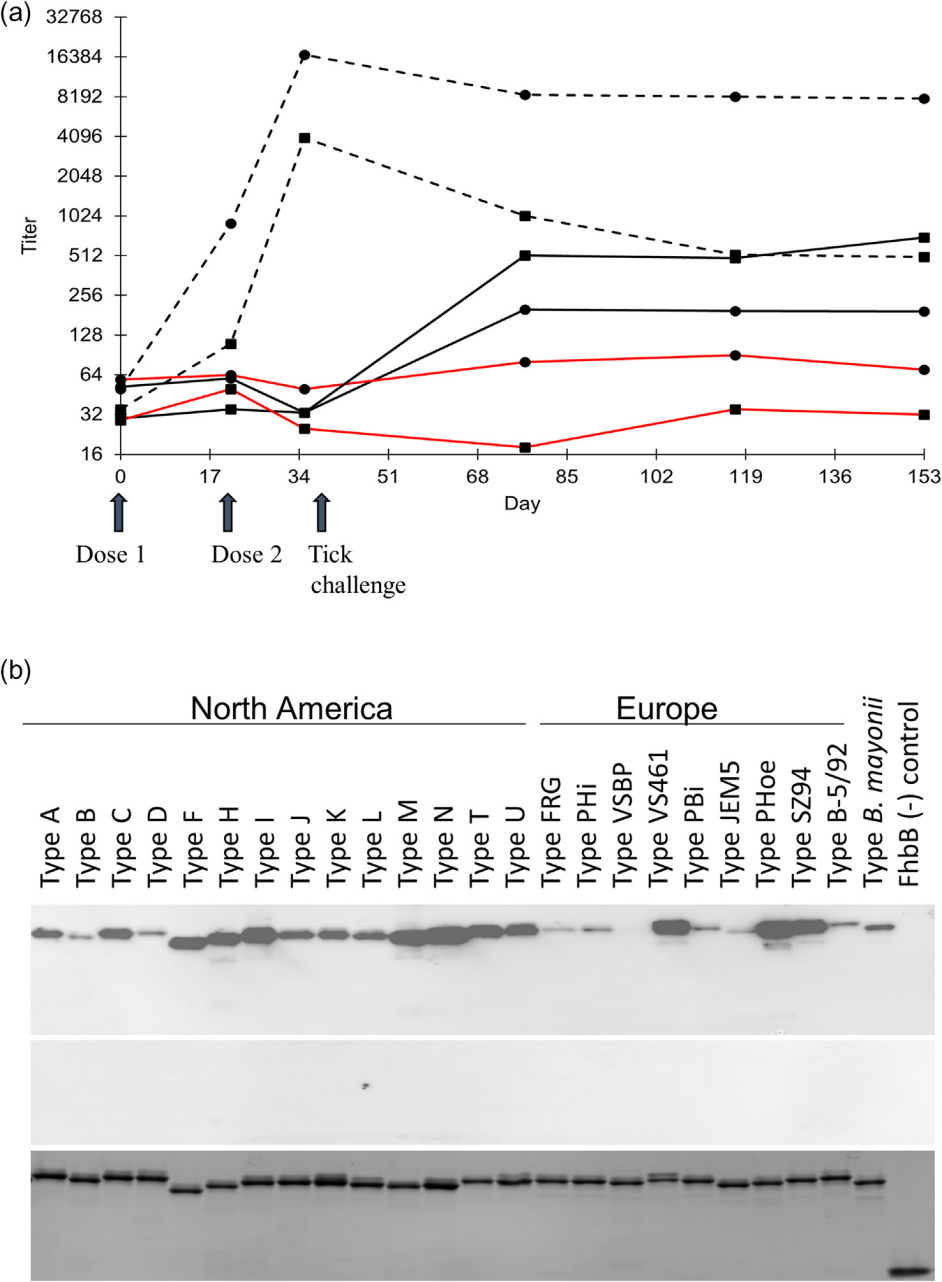
OspA and OspC (Ch14) Ab titers and cross-reactivity of vaccinal antibody with diverse OspC proteins. Geometic mean Ab titers were determined by ELISA using OspA and the Ch14 chimeritope proteins that constitute VANGUARD®crLyme as the immobilized detection antigens. Logarithm_2_ transformed antibody titers were analyzed with a general linear mixed model for repeated measures. The geometric mean titers at each time point for each study group are presented in the top panel. Placebo, vaccinate, and sentinel study groups are indicated by solid, dashed, and red lines, respectively. OspA data are indicated by black boxes (▪) and OspC data by black circles (●). Panel B presents a representative immunoblot in which serum from a VANGUARD®crLyme vaccinated dog was screened against twenty-five different OspC proteins (top image). The designations above each lane indicate the OspC type in each lane and its geographic origin. An identical blot was screened with preimmune sera (middle panel). The bottom panel is a coomassie stained gel that demonstrates similar loading for all proteins. The vaccinate and preimmune sera were used at a dilution of 1:5000. The *B. mayonii* OspC protein was cloned from a isolate collected a LD patient in in North America. The FhbB protein of *Treponema denticola* served as a negative control antigen. (For interpretation of the references to colour in this figure legend, the reader is referred to the web version of this article.)

Ab titers to the OspA and Ch14 were determined for each individual dog (Table 2). All vaccinates were Ab (+) for OspA and Ch14 on Day 35. The average Ab titers for each study group to each protein were determined over time (Fig. 2A). Non-specific Ab titers to Ch14 and OspA in the sentinel dogs reached maximal geometric means on Day 117 of 59.5 and 141.4, respectively. Non-specific Ab titers to Ch14 and OspA in placebo dogs prior to immunization and tick feeding reached geometric maximal means on Day 21 of 42.0 and 70.7, respectively. The highest geometric mean Ab titer to OspA (19740.3) and Ch14 (4935.1) in the vaccinates was measured on Day 35, 14 days after the second and final vaccine dose. OspC seroconversion was evident in the placebo dogs 25 days after the tick feeding (Day 77). OspA titers for the placebo group on Day 35 and 77 were 32.4 and 270.9. The titer data are consistent with the known expression patterns of OspC and OspA in mammals [47].

**Table 2:** Measurement of Ab titers to OspA and OspC (Ch14).

	Day of Study – OspA/OspC Titers					
Placebo(ID)	0	21	35	77	117	153
SER2	50/25	100/100	100/400	400/3200	800/400	800/1600
VGR2	<25/25	25/25	<25/<25	100/800	100/400	100/400
VIQ2	<25/<25	25/<25	<25/<25	200/800	400/1600	200/1600
VKQ2	50/25	100/25	100/50	400/400	400/200	400/200
VXQ2	100/25	200/25	50/25	200/6400	200/1600	200/400
WBQ2	50/25	50/<25	<25/<25	400/400	400/200	400/400
WCQ2	400/25	200/100	50/25	200/400	400/800	400/800
WFQ2	50/25	100/100	<25/50	800/800	800/3200	400/1600
XAR2	200/<25	200/50	25/25	400/400	400/400	400/800
XCR2	25/25	<25/50	<25/50	200/200	100/100	100/100
XFQ2	25/<25	25/50	<25/100	200/3200	400/400	400/800
XKR2	100/50	50/25	50/25	800/800	200/400	100/400
XLR2	100/50	100/25	100/100	100/800	50/400	100/1600
XPQ2	<25/25	200/200	200/50	400/800	800/400	400/800
YAQ2	100/25	100/25	25/25	400/1600	200/800	200/3200
YDR2	25/25	50/100	25/50	100/800	50/400	100/400

**Vaccinate ID**	**0**	**21**	**35**	**77**	**117**	**153**
SJQ2	100/100	200/100	6400/1600	3200/200	3200/400	1600/200
VFR2	25/25	1600/50	25600/3200	6400/400	6400/200	6400/100
VHR2	<25/<25	400/100	6400/6400	3200/1600	3200/800	3200/400
VJQ2	50/25	3200/200	51200/1600	12800/800	12800/400	12800/200
VTR2	100/25	3200/800	25600/25600	12800/6400	6400/3200	12800/1600
VYR2	200/25	1600/200	51200/12800	12800/3200	12800/1600	12800/1600
WAR2	100/100	6400/400	51200/12800	12800/6400	12800/3200	6400/3200
WEQ2	25/<25	1600/100	6400/6400	6400/400	3200/400	12800/200
WZR2	<25/25	1600/200	25600/12800	12800/6400	6400/400	6400/800
XDQ2	50/50	400/100	25600/6400	800/800	1600/400	800/400
XGQ2	100/50	1600/200	51200/6400	6400/800	6400/800	1600/400
XIR2	100/50	100/100	6400/1600	3200/400	3200/200	3200/200
XJR2	100/100	100/100	12800/3200	3200/400	3200/400	3200/200
XOQ2	25/25	200/100	12800/800	3200/200	3200/400	3200/200
YBQ2	25/50	800/200	25600/6400	6400/400	6400/400	3200/400
YFR2	25/25	800/100	25600/6400	12800/800	6400/800	6400/400

**Sentinel ID**	**0**	**21**	**35**	**77**	**117**	**153**
TBQ2	100/25	100/100	50/50	100/25	50/50	50/50
WDQ2	25/50	50/25	50/<25	100/<25	200/50	100/50
XBR2	50/25	50/25	50/25	200/25	200/50	200/50
XXR2	50/<25	100/50	50/25	100/25	200/100	100/<25

Vaccinations were on days 0 and 21 and tick challenge spanned days 42–51.

To determine if vaccination with VANGUARD®crLyme induces broadly cross-reactive antibodies to diverse OspC proteins, immunoblots consisting of twenty-five recombinant OspC variants were screened with sera collected on Day 0 and Day 35. All OspC variants tested were immunoreactive with sera from vaccinate dogs (Fig. 2B).

### 3.3 Histopathology

**Fig. 3:**
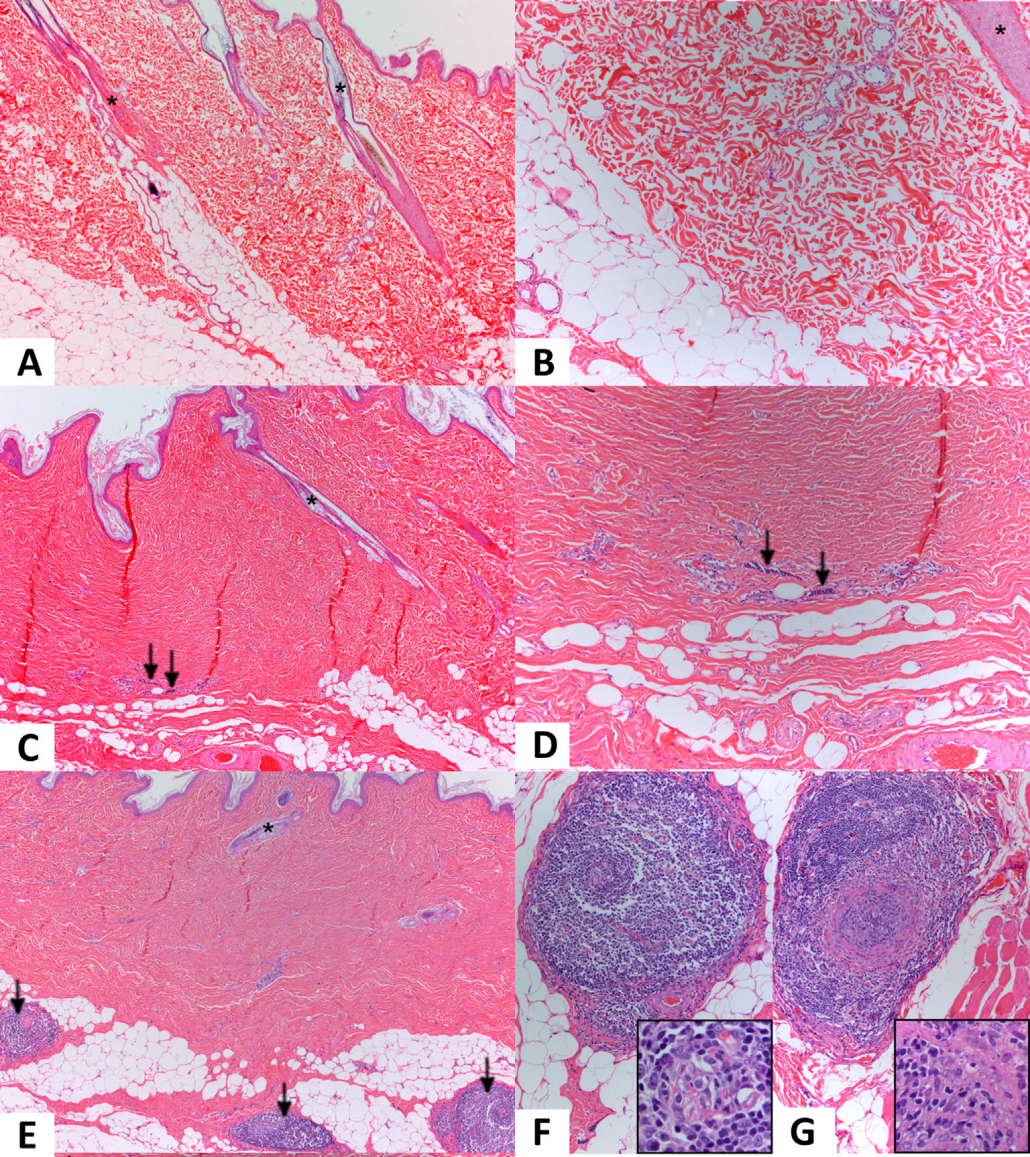
Histological changes seen at tick bite sites. Representative images from the skin of sentinel (A, B), vaccinate (C, D), and placebo (E, F, G) dogs are shown. Hair follicles are identified by asterisks. Areas of hair loss and subsequent dermal collagen condensation are evident in skin collected from the tick bite site in both vaccinate (C) and placebo (E) dogs. Mononuclear inflammation at the dermal-subcutis junction is indicated by the arrows in panel C and at higher magnification in panel D; note the condensed dermal collagen. For reference, healthy deep dermis exhibiting loose bundles of collagen and subcutaneous fat in a sentinel dog are shown in panels A and B. In a placebo dog (E) pronounced areas of nodular inflammation in the subcutis are indicated by arrows. Panels F and G are higher magnifications of the nodular subcutaneous areas in placebo dogs, and demonstrate that the mononuclear inflammation is centered on nerves (F) and blood vessels (G). The insets in F and G provide greater detail of the perineuritis and vasculitis, respectively. Note that the inflammation seen in the vaccinate (C, D) is typical for a tick bite site, whereas the nodular inflammation in the placebo dog (E) is characteristic of *B. burgdorferi* infection. Panels A, C, and E were photographed at 40X, B, D, F, and G at 100X, and the insets of F and G at 400X.

**Fig. 4:**
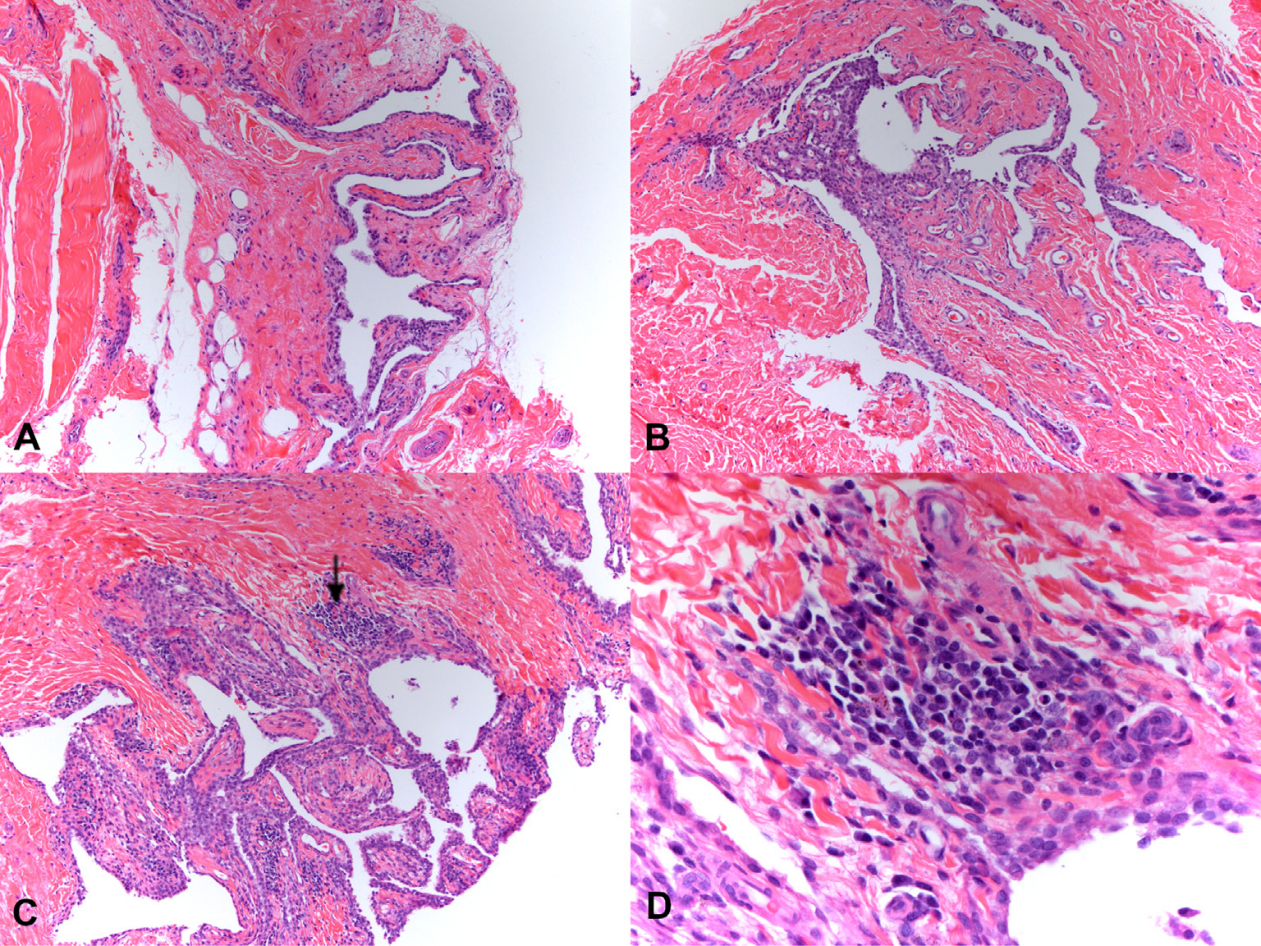
Histological changes seen in joints. Representative images from joints of sentinel (A), vaccinate (B), and placebo (C, D) dogs are shown. Sentinel (A) and vaccinate (B) joints exhibited mild synovial hyperplasia and minimal (A) to mild (B) mononuclear inflammation (as increased cellularity at this magnification). In contrast, there was an increase in both synovial hyperplasia and nodular inflammation in placebo dogs (C); the inflammation was lymphoplasmacytic (D). Panels A, B, and C were photographed at 200X, and Panel D at 400X.

Histopathology changes were noted at the tick bite sites and within and surrounding joints among study groups (Fig. 3, Fig. 4). At tick bite sites, placebos, vaccinates, and sentinels differed in the distribution of mononuclear inflammation (Fig. 3). Nodular lymphoplasmacytic inflammation typical of *B. burgdorferi* was present in the hypodermis/subcutis of 14/16 placebo dogs , but was absent from the hypodermis/subcutis of vaccinates and sentinels. Mononuclear inflammation with decreased numbers of hair follicles (alopecia) and subsequent dermal collagen condensation was identified in 10/16 placebos, 6/16 vaccinates, and 0/4 sentinels. Results are summarized in Table 1. Additionally, mononuclear inflammation at the tick bite sites often surrounded nerve fibers (perineuritis) and/or blood vessels (perivasculitis and vasculitis).

Within and around joints, histopathological changes included synovial hypertrophy and hyperplasia (including papillary projections), mononuclear and neutrophilic inflammation within the joint and local subcutis, and fibrin deposition (Fig. 4). Synovial hypertrophy and hyperplasia were characterized by an increase in the size and number of synovial cells. Intra-articular lymphoplasmacytic inflammation of Grade 2 or higher (“abnormal”) was identified in 13/16 placebos, 0/16 vaccinates, and 0/4 sentinels. As Grade 1 or 0 (“normal”) mononuclear inflammation was present in 0/16 placebos, 9/16 vaccinates, and 3/4 sentinels, this quantity of mononuclear inflammation was concluded to be within normal limits. Inflammation was commonly identified in more than one joint of placebo dogs. Vaccination with VANGUARD®crLyme prevented the synovitis and dermatitis typically associated with *B. burgdorferi* infection (p < 0.0001).

### 3.4 Determination of disease status

Dogs were scored as (+) for LD if they seroconverted to C6 Ab and met one of the following clinical criteria: 1) stiffness, limping, or lameness on three or more consecutive days or three or more events of intermittent lameness; 2) multiple episodes of clinical signs suggestive of *B. burgdorferi* infection; 3) abnormal histopathology. Based on these criteria, 16/16 placebo dogs were LD (+) while all sentinel dogs and vaccinates were LD (-). In summary, vaccination with VANGUARD®crLyme resulted in a significant difference (P < 0.0001) in the prevention of disease caused by *B. burgdorferi* (Table 1).

### 3.5 Vaccine safety

The temperature of each dog was monitored over time. One sentinel (not vaccinated) dog (XXR2) presented with a temperature of 39.9 °C on day 23. Two placebo dogs (SER2 and XKR2) had temperatures of 39.3 °C on three occasions. One vaccinate (SJQ2) had a temperature of 39.4 °C on Day 23. Elevated temperatures were not associated with the administration of placebo or vaccine. In summary, VANGUARD®crLyme is safe when administered subcutaneously as a two-dose series (three weeks apart) starting at approximately 8 weeks of age.

## 4 Discussion

VANGUARD®crLyme is a USDA approved canine LD vaccine. It is a subunit vaccine consisting of non-lipidated OspA and an OspC chimeritope (referred to Ch14). As detailed above, Ch14 is a recombinant protein consisting of seven L5 and seven H5 OspC epitopes from diverse OspC type proteins. This design strategy allows for the removal of conserved segments of OspC that do not elicit protective Ab responses and the generation of a protein consisting primarily of well-characterized linear epitopes from diverse OspC types [43], [44], [45], [46]. The rationale for the inclusion of both OspA and Ch14 was to develop a vaccine formulation that elicits Ab responses that can target diverse *B. burgdorferi* strains in both ticks and mammals. In this study, we demonstrated the immunogenicity and broad protective efficacy of VANGUARD®crLyme in a purpose bred dog-*Ixodes scapularis* challenge model.

In a recent study, Grosenbaugh et al [48] compared the anti-OspA Ab responses induced by vaccination of purpose bred dogs with RECOMBITEK® Lyme (Boehringer Ingelheim) and VANGUARD®crLyme (Zoetis). Both vaccines triggered high-titer Ab responses after the second dose that remained equivalent at multiple time points out to 16 weeks after the second vaccine dose [48]. The OspA in these vaccines differ in that RECOMBITEK® Lyme contains a lipidated form of OspA whereas the OspA of VANGUARD®crLyme is not lipidated. While Ab responses to OspC were assessed in that study, the results are compromised because a single monovalent OspC, and not the multi-valent OspC chimeritope (Ch14) that is an antigen in VANGUARD®crLyme, was used as the detection antigen. The immunogenicity of OspC chimeritopes has been demonstrated in other studies [43], [44], [45], [46]. To confirm and verify the immunogenicity of both vaccinogens that comprise VANGUARD®crLyme, antigen-specific IgG titers were determined. OspA and Ch14 elicited significant IgG titers in all vaccinates 3 weeks after the first vaccine dose with peak titers measured 14 days after the second dose (Day 35) (Fig. 2). .

To assess protective efficacy, placebo and vaccinates were challenged using field collected ticks. The use of field collected ticks, as opposed to laboratory infected ticks, is important for several reasons. First, the use of naturally infected ticks circumvents the inherent limitations associated with the use of ticks infected with laboratory adapted strains. Second, field collected ticks typically carry a heterogenous population of *B. burgdorferi* strains that produce different OspC types [40], [49]. Rhodes et al [50] amplified and sequenced a diverse array of *ospC* types from tissue and tissue derived cultures from dogs infected using field collected ticks. One of the most frequently amplified OspC types in that study was OspC type F. OspC type F specific antibody responses have been detected in horses and wild canids (eastern coyotes) [25], [38]. This OspC type has not to our knowledge been recovered from humans. Hence, the use of field collected ticks allow for an assessment of broad protective efficacy. The primary readouts of protective efficacy were the absence of development of Ab to the C6 peptide, prevention of LD associated clinical manifestations (lameness and joint inflammation), and prevention of abnormal histopathology of tissues and joints.

All dogs that received the placebo seroconverted to the C6 peptide after tick challenge while fifteen of sixteen VANGUARD®crLyme vaccinated dogs were C6 Ab (-) at all time points. One vaccinate was C6 Ab (+) at a single timepoint. However, that dog was C6 Ab (-) at all other timepoints and did not develop clinical or histopathological changes suggestive of LD. Vaccination with VANGUARD®crLyme also prevented the development of clinical manifestations commonly associated with LD. Consistent with earlier studies, some of the placebo dogs developed clinically apparent disease with single or multiple episodes of lameness post-tick feeding [40], [51]. Since overt clinical manifestations of LD typically develop slowly, tissues and joints were assessed for subclinical indications of infection. Increased lymphoplasmacytic inflammation was noted at 87.5% of tick bite sites and 81.3% of joints in placebo dogs, but none of the vaccinate or sentinel dogs. The absence of dermatitis and synovitis and other histopathological abnormalities in the vaccinates indicates that VANGUARD®crLyme provided significant protection (*P* < 0.0001) against *B. burgdorferi* infection. Importantly, vaccination did not result in adverse events demonstrating that VANGUARD®crLyme is safe for use in dogs 8 wks of age or older.

As discussed above, Ab to both OspA and OspC can target *B. burgdorferi* in ticks and inhibit transmission to mammals. Consistent with this, Ab titers to OspA and OspC did not increase in the VANGUARD®crLyme vaccinated dogs after tick feeding. If transmission had occurred, an increase in Ab titers to OspC (as measured using Ch14) would have been expected due to the expression of OspC by *B. burgdorferi* in mammals [38]. As expected, OspC IgG titers in the placebo dogs rose after tick feeding and remained elevated through the end of the study.

Previous studies revealed that immunization with prototype OspC chimeritopes triggers antibody responses to all OspC types represented in the vaccine construct [43], [44], [45], [46]. To determine if the Ch14 chimeritope in VANGUARD®crLYME induces broadly cross-reactive antibodies to OspC, immunoblot analyses were performed in which twenty-five different OspC variants were screened with sera from vaccinate dogs. The OspC types selected for screening included several from North America and Europe (Fig. 2B). All OspC types, including ones not directly represented in the chimeritope such as those from European isolates and *B. mayonii* [52], were immunoreactive with vaccinal antibody. In light of the fact that the LD spirochete population in a single tick can be heterogenous and produce as many as 21 different OspC types [53], the ability to elicit cross-reactive antibody is critical for providing broad protective efficacy. VANGUARD®crLyme is the only LD vaccine that has been directly demonstrated to elicit Ab against diverse OspC types. While it is stated by the vaccine manufacturer (Zoetis) that the vaccine contains epitopes from seven OspC types, the Ch14 chimeritope is clearly able to trigger broad antibody responses against a wider array of OspC type proteins. Comparative analyses of L5 and H5 epitope sequences have identified regions of amino acid identity between some OspC types [36]. It is likely that Ab elicited against these antigenic determinants is able to bind to other OspC types. The ability of vaccinal antibody to recognize diverse OspC types derived from European LD strains and *B. mayonii* raises the possibility that the vaccine could potentially protect against newly emerging species in North America and LD species and strains present in Eurasia. This remains to be demonstrated.

VANGUARD®crLyme was specifically designed to provide protection through two synergistic mechanisms of action. Vaccination induced Ab to OspA can target spirochetes in the tick [54] while Ab to OspC can target spirochetes in both ticks and mammals [49]. Here we demonstrated that the OspA and Ch14 (OspC) components of the vaccine are immunogenic and elicit protective Abs. No adverse vaccination events were observed, indicating the vaccine is safe. VANGUARD®crLyme provided significant prevention against *B. burgdorferi* infection and sub-clinical arthritis. As described in the 2018 American College of Veterinary Internal Medicine Lyme Borreliosis consensus statement, VANGUARD®crLyme is the only canine LD vaccine on the market with a USDA-approved 15-month duration of immunity label [55]. VANGUARD®crLyme provides the veterinary community with an effective preventative tool for combating LD in canines.

## Declaration of Competing Interest

Richard T Marconi is an inventor of VANGUARDcrLyme and a consultant for Zoetis. RTM receives royalties and speaking fees from Zoetis. All other authors were employees of Zoetis at the time the studies were conducted.

## Footnotes

1 https://capcvet.org/maps/#2020/all/lyme-disease/dog/united-states/

2 http://www.cvbd.org/en/tick-borne-diseases/lyme-borreliosis/distribution/
